# Genomic Insights into Blood Pressure Regulation: Exploring Ion Channel and Transporter Gene Variations in Jordanian Hypertensive Individuals

**DOI:** 10.3390/medicina61010156

**Published:** 2025-01-17

**Authors:** Mansour Abdullah Alghamdi, Laith AL-Eitan, Rasheed Ibdah, Islam Bani Khalid, Salma Darabseh, Maryam Alasmar, Asaad Ataa

**Affiliations:** 1 Department of Anatomy, College of Medicine, King Khalid University, Abha 62529, Saudi Arabia; 2 Genomics and Personalized Medicine Unit, College of Medicine, King Khalid University, Abha 62529, Saudi Arabia; 3 Department of Biotechnology and Genetic Engineering, Jordan University of Science and Technology, Irbid 22110, Jordanasaad.ataa@tu.edu.ye (A.A.); 4 Internal Medicine Department, College of Medicine, Jordan University of Science and Technology, Irbid 22110, Jordan

**Keywords:** hypertension, polymorphism, SNPs, WNK1

## Abstract

*Background and Objectives*: Hypertension (HTN) constitutes a significant global health burden, yet the specific genetic variant responsible for blood pressure regulation remains elusive. This study investigates the genetic basis of hypertension in the Jordanian population, focusing on gene variants related to ion channels and transporters, including *KCNJ1*, *WNK1*, *NPPA*, *STK39*, *LUC7L2*, *NEDD4L*, *NPHS1*, *BDKRB2*, and *CACNA1C*. *Materials and Methods*: This research involved 200 hypertensive patients and 224 healthy controls. Whole blood samples were collected from each participant, and genomic DNA was extracted. The genetic distribution of the polymorphisms was analyzed. The haplotype frequencies were investigated using the SNPStats web tool, and the genotype and allele frequencies of the studied variants were assessed using the χ^2^ test. *Results*: Sixteen single nucleotide polymorphisms (SNPs) from nine genes were evaluated. A significant association was observed between the rs880054 variant of the *WNK1* gene and hypertension susceptibility, with the T allele elevating the risk of hypertension. This association remained important in the codominant model (*p* = 0.049) and the dominant model (*p* = 0.029). In addition, rs880054 was associated with clinical characteristics such as triglyceride levels and cerebrovascular accidents (*p*-value > 0.05). *Conclusions*: Our findings reveal a significant link between the rs880054 SNP and an increased hypertension risk, suggesting that variations in *WNK1* may be crucial in regulating blood pressure. This study provides new insights into the genetic factors contributing to hypertension and highlights the potential of WNK1 as a target for future therapeutic interventions.

## 1. Introduction

A billion people worldwide suffer from hypertension (HTN), which is a significant modifiable risk factor for heart disease and mortality [1,2,3,4,5,6,7,8]. Due to its high prevalence and the associated increased risk of vascular diseases, HTN is a global public health issue [9]. According to studies, HTN is a polygenic, complex condition influenced by genetic factors (accounting for 30–60%) and environmental variables such as lifestyle (e.g., obesity, excessive salt consumption, and lack of physical exercise), living circumstances, and dietary factors [2,7,10,11,12]. However, the cause of HTN and the underlying factors contributing to inter-individual blood pressure variation in over 90% of individuals remain unclear [2,10]; thus, identifying the candidate genes involved in the mechanisms regulating blood pressure across diverse populations is crucial for preventing and treating HTN [2,9,10,11].

A proposed hypothesis suggests that genetic variants affecting ion channels, transporters, and regulatory proteins involved in salt and water reabsorption may constitute the fundamental basis of HTN [11]. STK39, which stands for serine/threonine kinase 39, is a kinase with structural similarity to sterile 20-like kinases, which is rich in proline and alanine residues. It plays a crucial role in the phosphorylation of various cation–chloride-coupled cotransporters, thereby contributing to maintaining the salt-water balance. Additionally, STK39 interacts with p38 MAP kinase pathway components, mitigating cellular stress-induced damage [7,13]. The *STK39* gene sequence is polymorphic, and 34 variants within the *STK39* gene have been identified as being associated with hypertension [7,8,13,14,15].

WNK1 (with no lysine kinase 1) is a serine/threonine kinase that regulates several ion channels involved in sodium and potassium transport [9]. *WNK1* is located on chromosome 12p13.3, consists of 29 exons, and spans approximately 150 kb [9,16,17]. *WNK1* is highly polymorphic, with over 100 verified single nucleotide polymorphisms [4,18]. It is ubiquitously expressed throughout the body, with particularly high expression levels observed in the kidney and cardiovascular system [5,19]. WNK1 has been recognized as a pivotal regulator of salt homeostasis, controlling the balance of renal sodium reabsorption and potassium excretion [5,9,19,20]. Several studies have highlighted the involvement of various common *WNK1* gene variants, such as rs880054, in blood pressure variation within the general population [4,16,17,18]. Therefore, further research is required to identify specific regions of the *WNK1* gene that harbor functional genetic variations associated with hypertension (HTN) and medication response [4,17,21].

*NEDD4L* (neural precursor cell-expressed developmentally down-regulated 4-like) is another essential gene that encodes a Ste-related proline–alanine-rich kinase [3,10,14]. NEDD4L is an E3 ubiquitin ligase enzyme that regulates epithelial Na nephron cell surface expression [3,22,23]. Many studies have shown that the *NEDD4L* gene polymorphisms may influence hypertension by inhibiting the epithelial sodium channel (ENaC—amiloride-sensitive epithelial Na^+^ channel) through ubiquitination during increased salt intake [3,14,15,23,24,25]. The ENaC is located in the distal nephron and is primarily responsible for regulating sodium and body fluid reabsorption and facilitating sodium entry from the renal lumen into cells [10,22,23,24,26].

In response to cardiac output overload, the natriuretic peptide system, with atrial natriuretic peptide (ANP) as a key component, plays a crucial role in maintaining the salt–water balance and blood pressure through mechanisms such as diuresis, natriuresis, and vasodilation [6]. Atrial natriuretic peptide (ANP) has a significant role in the development of hypertension [6,27]. Indeed, there is a well-established and compelling link between atrial natriuretic peptide (ANP) and hypertension [6,27].

Given the above, the purpose of this investigation was to examine the occurrence and potential relationship between candidate gene variants such as *KCNJ1* (rs675388, rs59172778, rs12795437, and rs11600347), *WNK1* (rs880054), *NPPA* (rs5065), *STK39* (rs6749447), *LUC7L2* (rs6947309), *NEDD4L* (rs75982813, rs292449), *NPHS1* (rs3814995), *BDKRB2* (rs1799722, rs8012552), and *CACNA1C* (rs2239128, rs2238032, rs1051375) and hypertension in the Jordanian Arab population.

## 2. Methods

### 2.1. Participants

This study included 224 healthy controls and 200 associated hypertension patients from the Jordanian Arab community, recruited from the Cardiac Clinic and Coronary Cardiac Care Unit at King Abdullah University Hospital (KAUH) in Irbid, Jordan. These patients have cardiovascular conditions, such as high blood pressure, and are being treated with antihypertensive medications from various classes. All the participants in this study were 35 or older and had been on antihypertensive medications for at least a year. Demographic and clinical information was collected using KAUH’s electronic medical records system. All the participants provided written informed consent. The study protocol was approved by the Human Ethics Committee of Jordan University of Science and Technology and King Abdullah University Hospital (KAUH) (Date: 29 July 2020, No.: 4/133/2020).

Out of a total population of 10,699,000 in Jordan, 1.3 million adults between the ages of 30 and 79 have hypertension, representing a prevalence of 12%, according to the World Health Organization (WHO) (https://www.who.int/publications/m/item/hypertension-jor-2023-country-profile, accessed on 1 November 2024). The OpenEpi software, version 3.01, was used to determine the sample size with a 95% confidence interval. With a precision of 5%, a design effect of 1, and a prevalence of 12% for hypertension in Jordan, the sample size was determined to be 163 patients. For the cases in our study, 200 participants represented the sample size. After the exclusion criteria were applied, 320 patients initially screened were reduced to 200, exceeding the required sample size.

### 2.2. Gene and SNP Selection

In the current study, 16 SNPs located in the candidate genes *KCNJ1*, *WNK1*, *NPPA*, *STK39*, *LUC7L2*, *NEDD4L*, *NPHS1*, *BDKRB2*, and *CACNA1C* were selected due to their relevance to hypertension susceptibility. The SNPs were selected using publicly available databases, including the National Center for Biotechnology Information (NCBI) SNP database (http://www.ncbi.nlm.nih.gov/SNP/, accessed on 1 November 2024) and the Ensembl database (http://www.ensembl.org/index.html, accessed on 1 November 2024). To explore the functional annotations of the variants analyzed in this work, we utilized the resources provided by HaploReg v4.2 (https://pubs.broadinstitute.org/mammals/haploreg/haploreg.php, accessed on 1 November 2024) and RegulomeDB version 2.2 (https://regulomedb.org/regulome-search/, accessed on 1 November 2024).

The functional annotation using HaploReg v4.2 revealed that rs11600347, rs12795437, rs880054, rs6947309, rs6749447, rs8012552, rs5065, rs3814995, rs2238032, and rs2239128 are intronic variants. Rs1051375 is a synonymous variant, while rs675388, rs292449, rs1799722, and rs75982813 are upstream transcript variants. Additionally, rs59172778 is classified as a missense variant. These SNPs influence enhancer histone marks and alter transcription factors’ binding motifs, which may indirectly influence hypertension pathogenesis by modulating the transcriptional activity of the relevant genes. Based on their regulatory relevance, the SNPs chosen for this study were selected using the RegulomeDB database. Notably, many selected SNPs are ranked as 1a, indicating strong evidence of regulatory function, such as involvement in gene expression (eQTLs), transcription factor binding, and chromatin interactions. This ranking reflects their functional relevance and biological significance, making them promising candidates for investigating the genetic mechanisms contributing to disease susceptibility.

### 2.3. Extraction and Genotyping of Genomic DNA

Blood samples were obtained from all the participants following ethical guidelines and proper consent procedures. Genomic DNA extraction was performed using the QIAGEN Blood DNA Mini Kit (QIAGEN, Germantown, MD, USA) (Cat. No. 51106) per the manufacturer’s protocol. To assess the quantity and quality of the extracted DNA, spectrophotometric analysis was conducted using a BioDrop (BioDrop Ltd., Cambridge, UK), which allowed for the measurement of absorbance at 260 nm and 280 nm to determine the DNA concentration and purity. A ratio of A260/A280 close to 1.8–2.0 indicated high-quality DNA. Additionally, the DNA integrity was also assessed by gel electrophoresis on a 1% agarose gel using the gel electrophoresis system (Cleaver Scientific Ltd., Rugby, Warwickshire, UK). This method allowed for the visualization of the DNA quality, with intact genomic DNA typically exhibiting a sharp, high-molecular-weight band and minimal degradation. Only DNA samples with sufficient concentration, purity, and integrity were selected for the downstream genetic analyses. The Luminex DNA array technique was used at the Australian Genome Research Facility to genotype the examined gene variants according to the manufacturer’s guidelines (AGRF; Melbourne Node, Melbourne, Australia). A total of sixteen single nucleotide polymorphisms (SNPs) within nine genes were analyzed in this study (*KCNJ1* (rs675388, rs59172778, rs12795437, and rs11600347), *WNK1* (rs880054), *NPPA* (rs5065), *STK39* (rs6749447), *LUC7L2* (rs6947309), *CACNA1C* (rs2239128, rs2238032, rs1051375), *NEDD4L* (rs75982813, rs292449), *NPHS1* (rs3814995), and *BDKRB2* (rs1799722, rs8012552). Table 1 presents details of the selected genes and their SNPs, including the chromosomal positions and functional annotations, in the study cohort.

### 2.4. Statistical Analyses

The statistical analyses were performed by employing multiple methods and software tools to investigate the genotype–phenotype relationship and evaluate the association of genetic variants with disease susceptibility. The genotype–phenotype interactions were assessed using Pearson’s chi-square test and one-way ANOVA, and odds ratios (ORs) with 95% confidence intervals (CIs) were computed to estimate the risk. One-way ANOVA was utilized as the data were continuous, and the analysis was designed to assess differences in the means across multiple groups. The data analyses were performed using the Statistical Package for Social Sciences (SPSS) version 26.0 (SPSS, Inc., Chicago, IL, USA). Furthermore, SNPStat (https://www.snpstats.net/start.htm, accessed on 1 November 2024) was utilized to investigate the inheritance models, test the Hardy–Weinberg equilibrium, estimate the haplotype frequencies, and explore the connection between the haplotypes and the disease risk. A *p*-value threshold of 0.05 was used to determine statistical significance. An adequate number of SNPs were assessed following the procedure described in [28] to correct multiple testing. Moreover, the Bonferroni adjustment was implemented by adjusting the significance threshold to α/n, with α = 0.05 and n indicating the number of individual tests [29].

## 3. Results

### 3.1. General Characteristics and Hardy–Weinberg Equilibrium (HWE) Test

This study included 224 healthy controls with a mean age of 34.50 ± 12.44 years, 61.7% male. In comparison, there were 200 hypertension (HTN) patients with a mean age of 58.84 ± 10.39 years, 57.5% of whom were male, as shown in Table 2. Differences in age and body mass index (BMI) were observed between the control group and the HTN patients (*p* = 0.001, respectively). However, no significant differences were observed in other characteristics. The clinical characteristics of the patients included in this study are presented in Supplementary Table S1. The observed genotype distribution in the studied population was found to be in accordance with the Hardy–Weinberg equilibrium (HWE) (*p*-value > 0.05), as indicated in Table 3.

### 3.2. Genotypic Distribution and Genetic Model Analysis of Polymorphisms with HTN

The genotype and allele frequency distributions, as well as the genotype models of the studied polymorphisms in the study groups, as presented in Table 4 and Table 5, showed no significant differences (*p* > 0.05) for all the studied SNPs—KCNJ1 (rs675388, rs59172778, rs12795437, and rs11600347), NPPA (rs5065), STK39 (rs6749447), CACNA1C (rs2239128, rs2238032, rs1051375), LUC7L2 (rs6947309), NEDD4L (rs75982813, rs292449), NPHS1 (rs3814995), and BDKRB2 (rs1799722, rs8012552)—between the healthy controls and the hypertensive patients in the Jordanian Arab population, except for WNK1 (rs880054). For WNK1 (rs880054), the TT genotype was more frequent in the hypertensive patients (*p* = 0.049). A significant difference was detected between the healthy controls and the patients when comparing the homozygous dominant (CC) genotype with the homozygous recessive (TT) and heterozygous (CT) genotypes in the dominant model (*p* = 0.029, OR = 1.65). The T allele was significantly associated with hypertension (49%) compared to the healthy controls (40%) (*p* = 0.01). After applying the Bonferroni correction with a *p*-value threshold of 0.003, no significant genetic associations were identified between the SNPs and hypertension. A summary of the genetic association analysis results for hypertension susceptibility is presented in Table 6.

### 3.3. Genotype–Phenotype Association Analysis of HTN Patients

Supplementary Table S2 shows the relationship between the studied polymorphisms among the HTN patients and different clinical data. rs12795437 and rs11600347 of *KCNJ1* were significantly associated with chronic kidney disease (*p*-value = 0.0002), smoking (*p*-value = 0.03 and 0.014), HDL levels (*p*-value = 0.04), thiazide diuretics (*p*-value = 0.004), calcium channel blocker (CCB) (*p*-value = 0.005), immune-suppressive/allergies (*p*-value = 0.002), dialysis (*p*-value = 0.008), other stomach drugs (*p*-value = 0.003), white blood cell (WBC) count (*p*-value = 0.016 and 0.018), and total protein level (*p*-value = 0.02). rs675388 of *KCNJ1* was associated with ex-smoker status (*p*-value = 0.004), B-blocker (*p*-value = 0.002), angiotensin-converting enzyme inhibitor (ACEi) (*p*-value = 0.031), other HTN medications from other classes (*p*-value = 0.04), immune-suppressive/allergies (*p*-value = 0.033), ischemic heart disease (IHD) (*p*-value = 0.049), and left ventricular hypertrophy (LVH) on ECG (*p*-value = 0.025). rs6749447 of *STK39* was significantly associated with classes taken (*p*-value = 0.000), NSAID/COX2 (*p*-value = 0.000), other medications (*p*-value = 0.000), chemotherapy (*p*-value = 0.00), other stomach drugs (*p*-value = 0.000), WBC (*p*-value = 0.021), and cholesterol level (*p*-value = 0.032). rs880054 of *WNK1* was significantly associated with SBP (*p*-value = 0.027), glyceride level (*p*-value = 0.026), weight (*p*-value = 0.024), IHD (*p*-value = 0.039), cerebrovascular accident (*p*-value = 0.02), and atrial fibrillation (*p*-value = 0.02). rs6947309 of *LUC7L2* was associated with using other medications (*p*-value = 0.001). rs3814995 of *NPHS1* was associated with gender (*p*-value = 0.005), height (*p*-value = 0.011), IHD (*p*-value = 0.043), and use of antipsychotic medication (*p*-value = 0.001). rs292449 of *NEDD4L* was significantly associated with pulse rate (*p*-value = 0.002). rs75982813 of *NEDD4L* was linked with cardiovascular medications (*p*-value = 0.028) and angiotensin II receptor blocker (ARB) medications (*p*-value = 0.0001). rs1799722 of *BDKRB2* was significantly associated with HbA1c levels (*p*-value = 0.035), sodium (Na) level (*p*-value = 0.033), number of years with HTN (*p*-value = 0.046), ejection fraction (EF) (*p*-value = 0.03), and heart failure (*p*-value = 0.019). rs8012552 of *BDKRB2* was significantly associated with height (*p*-value = 0.003), LVH on ECHO (*p*-value = 0.03), total protein (*p*-value = 0.035), calcium channel blockers (CCBs) (*p*-value = 0.029), the number of HTN drugs from all classes (*p*-value = 0.02) and the number of required HTN drugs (*p*-value = 0.027). rs2239128 of *CACNA1C* was linked with several HTN drugs from all classes (*p*-value = 0.003) and the number of required HTN drugs (*p*-value = 0.008). *CACNA1C* rs2238032 exhibited significant relationships with creatinine clearance (*p*-value = 0.022), other medications for chest pain (angina) (*p*-value = 0.0001), B-blocker and thiazide diuretics (*p*-value = 0.003), and DM treatment (*p*-value = 0.006). rs1051375 of *CACNA1C* was linked with height (*p*-value = 0.034) and diet (*p*-value = 0.037).

### 3.4. Haplotype Analysis of the KCNJ1, NEDD4L, and BDKRB2 Genes

Haplotype analysis of the candidate genes *KCNJ1*, *NEDD4L*, *BDKRB2*, and *CACNA1C* was performed to investigate the potential associations with hypertension. However, the results revealed no significant associations between the identified haplotypes and hypertension for any of the genes studied. These findings are summarized in Table 7, where no haplotype was significantly associated with the disease risk.

## 4. Discussion

Despite the identification of numerous risk factors for hypertension (HTN), several decades of control efforts focused on interventions targeting these risk factors have proven unsuccessful in significantly improving the prevention and management of this global epidemic. The continued rise in hypertension suggests the presence of unidentified mechanisms contributing to its persistence [6]. A deeper understanding of the molecular processes linking blood-pressure-regulating genes to hypertension could help develop and improve new drugs and enhance treatment strategies [6]. In this study, we examined the associations of sixteen single nucleotide polymorphisms (SNPs) from nine genes (*KCNJ1* (rs675388, rs59172778, rs12795437, and rs11600347), *CACNA1C* (rs2239128, rs2238032, and rs1051375), *WNK1* (rs880054), *NPPA* (rs5065), *STK39* (rs6749447), *LUC7L2* (rs6947309), *NEDD4L* (rs75982813 and rs292449), *NPHS1* (rs3814995), and *BDKRB2* (rs1799722 and rs8012552)) with the risk of hypertension in the Jordanian population.

Certain rare Mendelian forms of hypertension pathogenesis have been linked to genetic mutations involved in sodium and water reabsorption [30]. WNK1, a serine-threonine kinase, regulates multiple ion channels involved in sodium and chloride transport within the kidney [31]. The sodium–chloride cotransporter (NCC) is a crucial channel for sodium reabsorption in the distal convoluted tubule (DCT), and its hyperactivity plays a significant role in promoting volume expansion and hypertension [32]. NCC reabsorption is regulated through direct phosphorylation by Ste20-like proline alanine-rich kinase (SPAK) and oxidative stress-responsive kinase 1 (OSR1), which are activated through phosphorylation by WNK proteins [33]. Mutations in the *WNK1* gene increase L-WNK1 expression in the distal tubule, leading to overstimulation of the WNK-SPAK/OSR1-NCC pathway, which results in enhanced NaCl reabsorption and contributes to hypertension [34].

WNK1 polymorphisms, which affect renal salt transport, influence blood pressure regulation and predict inter-individual variations in antihypertensive responses [35]. In the Han Chinese population, the rs880054 polymorphism showed a significant association with hypertension when comparing the allele frequencies, with a particular increased risk observed in males [36]. In a population-based sample of white European families, Tobin et al. found that rs880054 within *WNK1* gene is correlated with blood pressure variance [9]. Osada et al. recently demonstrated an association between the *WNK1* gene SNP rs880054 and blood pressure variations in the general Japanese population. Furthermore, they found that the resulting haplotypes were associated with the Na/K intake ratio [9]. This result is consistent with our previous finding of a significant association between the TT genotype and T allele of rs880054 and HTN (*p*-value = 0.047 and 0.0.2, respectively). The systolic blood pressure (SBP) and glyceride levels were associated with the TT genotype of rs880054 (*p*-value = 0.027 and 0.026, respectively). However, upon further examination of these promising results between rs880054 and blood pressure, it was noted that the correlation could be more consistent or nominal, as the same relationship was not replicated in another study [9].

The STK39 variants have been associated with hypertension by increasing STK39 mRNA expression, activating the SPAK–SLC12A signaling pathway. This activation results in Na-K-Cl cotransporter isoform 1 (NKCC1) phosphorylation, enhancing sodium chloride reabsorption in the kidneys and arteries. This cascade of molecular events increases susceptibility to hypertension [37].

In the current study, we focused on rs6749447 of *STK39*. The frequency of minor allele A of rs6749447 STK39 varies between populations. According to our findings, the A allele is present in 43% of Jordanians. In comparison, the prevalence of the minor A allele in the Chinese population was 23%, slightly lower than in the Caucasian cohort (28%) [13]. However, the frequency in a South African sample was notably high at 53% [11].

Several studies have found no association of the rs6749447 STK39 variants in the Iranian, Chinese, Korean, and British Caucasian populations [23,24,25], consistent with our findings in the Jordanian Arab population. *STK39* was identified as a potential hypertension susceptibility gene in a genome-wide association study (GWAS) conducted in the Amish population and subsequently validated in various non-Amish populations [8,11]. SPAK, which is involved in salt renal reabsorption and blood pressure regulation, is encoded by *STK39* [14]. Therefore, it is unsurprising that single nucleotide polymorphisms (SNPs) within *STK39* were discovered to be associated with hypertension through the genome-wide association studies (GWASs) [14]. We could not reproduce the associations between a common GWAS SNP, rs6749447, and HTN in the Jordanian community [11]. The underlying reality might be far more complicated, with numerous functional polymorphisms accounting for the total impact [11]—a recent replication. A study conducted in American Old Order Amish and two Swedish populations revealed that the functional polymorphism rs6749447 in *STK39* was correlated with blood pressure (BP) and hypertension [2]. In the Finnish population, the rs6749447 G allele was associated with hypertension, with this relationship observed in subjects followed until the age of 60 [38].

We also examined the relationships between two SNPs in *NEDD4L* (rs75982813, rs292449) and HTN [3]. However, we found no significant relationships between these variants and HTN in Jordanian populations; a great correlation with BP reduction was found in patients who had one or two copies of the rs4149601-rs292449 GC haplotype in the Chinese and Japanese populations [3,26,39]. rs292449 of *NEDD4L* had a significant relationship with the pulse rate (*p*-value = 0.002). rs75982813 of *NEDD4L* was linked with cardiovascular (*p*-value = 0.028) and angiotensin II receptor blocker (ARB) medications (*p*-value = 0.0001). rs8012552 of *BDKRB2* was significantly linked with total protein (*p*-value = 0.035), Na level (*p*-value = 0.033), number of years of HTN (*p*-value = 0.046), and heart failure (*p*-value = 0.019).

Since the identification of a family of vasodilatory and natriuretic hormones secreted by the heart in response to increased wall stress, it has been understood that these molecules, termed atrial natriuretic peptides (ANPs), are involved in the regulation of human blood pressure [1,6]. Elevated systolic blood pressure, induced by increased cardiac afterload, stimulates the synthesis of natriuretic peptides [1]. This protein is encoded by the natriuretic peptide A (*NPPA*) gene [1,6]. Previous research has shown that NPPA rs5068 functions in blood pressure control and the development of hypertension in the Japanese population, which contrasts with our findings [6,40]. For instance, a meta-analysis involving 4068 individuals revealed that the *NPPA* gene rs5065 SNP has been linked with hypertension, myocardial infarction, stroke, and coronary artery disease in the Chinese and Han populations [6,41]. Notably, the link between rs5068 at the *NPPA* gene and hypertension was significant genome-wide, with a *p*-value of 1 × 10^−8^ [6].

Voltage-dependent Ca^2+^ channels (VDCCs), responsible for regulating blood pressure by mediating Ca^2+^ entry into excitable cells, play a crucial role in various physiological activities, including vascular smooth muscle contraction [42]. The α-1a and α-1c subunits of voltage-dependent Ca^2+^ channels (VDCCs), which serve as targets for calcium channel blockers (CCBs), are encoded by the *CACNA1A* and *CACNA1C* genes [42]. Increased expression of VDCCs has been linked to Ca^2+^ imbalances and hypertension [42]. Therefore, it was hypothesized that the *CACNA1A* and *CACNA1C* genes were essential in the control of BP [34]. Nevertheless, no cohort study has specifically examined a single marker or combined relationships of the *CACNA1A* and *CACNA1C* genes with blood-pressure-related symptoms. However, relatively few investigations have been conducted within the Han Chinese population [42]. Our study and previous research found no association between CACNA1C (rs2239128, rs2238032, rs1051375) and HTN [42]. In contrast, rs2238032 has shown a significant association with uncontrolled hypertension in Caucasian populations [43,44]. In our study, CACNA1C rs2239128 was associated with weight (*p*-value = 0.014) and the number of HTN medications needed (*p*-value = 0.008). CACNA1C rs2238032 was found to have a significant relationship with creatinine clearance (*p*-value = 0.022), other medicines for chest pain (angina) (*p*-value = 0.0001), B-blocker and thiazide diuretics (*p*-value = 0.003), number of HTN drugs from all classes (*p*-value = 0.006), and diabetes treatment (*p*-value = 0.0001).

The results of replication experiments in various ethnic groups were inconsistent [7]. Genetic variability across races and ethnicities might be one factor [13]. Another explanation for these contradictory results is the modifiable influence of environmental or lifestyle factors on genetic predisposition [13]. While genetic factors may contribute to an individual’s susceptibility to a disease, the development of the disease is primarily influenced by exposure to specific environmental or lifestyle factors [13].

A limitation of the present study is the absence of functional validation for the identified single nucleotide polymorphisms (SNPs). While statistical associations are informative, the biological relevance of these genetic variants remains speculative without experimental validation. Functional studies, including gene expression analysis and pathway investigations, are essential to clarify the role of these SNPs in the pathophysiology of hypertension and their underlying molecular mechanisms. Additionally, the retrospective nature of this case-control study limits the ability to infer causality between these genetic variants and the development of hypertension. Longitudinal studies are necessary to establish temporal relationships and causative links. The generalizability of our findings should also be evaluated by replicating this study in more extensive, independent cohorts encompassing diverse populations. The limited sample size in this investigation underscores the need for further research with more comprehensive, rigorously designed studies to fully elucidate the causative mechanisms behind the observed associations. Genetic heterogeneity is a well-recognized challenge in genetic epidemiology, which can be partially addressed by focusing on more homogenous subgroups. Moreover, the potential influence of genetic variants may vary across different population groups. This necessitates studies assessing the impact of these SNPs in diverse racial and ethnic populations to understand their role in hypertension susceptibility.

## 5. Conclusions

In conclusion, the present study found no significant association between the selected genes and the risk of hypertension, except for rs880054 of the *WNK1* gene, in a population of Jordanian Arabs. Our findings hold considerable potential for broader applications, including developing genetic screening tools to identify individuals at higher risk of hypertension and designing targeted therapeutic strategies. These insights could contribute to personalized medicine approaches, enabling early intervention and tailored treatments based on an individual’s genetic profile. Future large-scale longitudinal studies investigating gene–gene and gene–environment interactions are recommended to explore the relationship between genetic variations and hypertension thoroughly.

## Figures and Tables

**Table 1:** Overview of the selected genes and their SNPs, chromosomal positions, and functional annotations in the study cohort.

Gene	SNP_ID	Position	SNP	Functional Annotation
***KCNJ1***	*rs11600347*	11:128863419	C>A ^MA^	Intron variant
	*rs12795437*	11:128860981	G>C ^MA^	Intron variant
	*rs59172778*	11:128839231	A>G ^MA^	Missense variant
	*rs675388*	11:128838114	G>A ^MA^	3′-UTR variant
***WNK1***	*rs880054*	12:879392	C ^MA^>A	Intron variant
***LUC7L2***	*rs6947309*	7:139351084	C>T ^MA^	Intron variant
***STK39***	*rs6749447*	2:168184876	T>G ^MA^	Intron variant
***NEDD4L***	*rs292449*	18:58227849	G>C ^MA^	5′-UTR variant
	*rs75982813*	18:58043776	A>G ^MA^	2KB_upstream_variant
***NPHS1***	*rs3814995*	19:35851310	C>T ^MA^	Intron variant
***BDKRB2***	*rs8012552*	14:96222430	C ^MA^>T	Intron variant
	*rs1799722*	14:96204802	C>T ^MA^	2KB_upstream_variant
***NPPA***	*rs5065*	1:11846011	A>G ^MA^	Intron variant
***CACNA1C***	*rs1051375*	12:2679713	G ^MA^>A	Synonymous variant
	*rs2238032*	12:2113566	T>G ^MA^	Intron variant
	*rs2239128*	12:2648603	T ^MA^>C	Intron variant

Chromosomal positions are based on the NCBI Human Genome Assembly Build. MA: Minor allele.

**Table 2:** Demographic data of the participants.

Category	Subcategory	Percentage (%)/Mean ± SD	
		Controls	HTN Patients
**Gender**	Male	61.7%	57.5%
	Female	38.3%	42.5%
**Age**	-----------	34.50 ± 12.44	58.84 ± 10.39
**BMI**	-----------	30.56 ± 56.26	33.35 ± 22.58
**Smoker**	Yes	44%	28%
	No	56%	72%

**Table 3:** The genes, their SNPs, their minor allele frequencies, and the HWE *p*-value in cases and controls.

Gene	SNP_ID	Cases (*n* = 200)			Controls (*n* = 224)		
		HWE ^c^ *p*-Value	MAF ^b^	MA ^a^	HWE ^c^ *p*-Value	MAF ^b^	MA ^a^
***KCNJ1***	*rs11600347*	1	0.07	A	0.6	0.08	A
	*rs12795437*	0.59	0.07	C	1	0.07	C
	*rs59172778*	Monomorphic SNP-------------------------------					
	*rs675388*	1	0.14	A	1	0.12	A
***WNK1***	*rs880054*	1	0.5	C=T	0.87	0.41	T
***LUC7L2***	*rs6947309*	0.75	0.34	T	0.17	0.34	T
***STK39***	*rs6749447*	0.46	0.43	G	0.31	0.35	G
***NEDD4L***	*rs292449*	0.77	0.46	C	0.64	0.48	C
	*rs75982813*	1	0.05	G	1	0.04	G
***NPHS1***	*rs3814995*	0.84	0.23	T	0.28	0.23	T
***BDKRB2***	*rs8012552*	0.55	0.41	C	0.26	0.38	C
	*rs1799722*	0.37	0.39	T	0.87	0.38	T
***NPPA***	*rs5065*	0.35	0.19	G	0.22	0.2	G
***CACNA1C***	*rs1051375*	0.67	0.48	G	0.44	0.47	A
	*rs2238032*	1	0.01	G	0.083	0.02	G
	*rs2239128*	0.36	0.38	T	0.88	0.43	T

^a^ MA: Minor allele. ^b^ MAF: Minor allele frequency. ^c^ HWE: Hardy–Weinberg equilibrium.

**Table 4:** Genotype and allele distributions of the SNPs within the genes in hypertensive patients and controls.

Gene	SNP_ID	Genotype/Allele	Frequency		*p*-Value
			Controls N (%)	Cases N (%)	
***NPPA***	*rs5065*	A/A	112 (67%)	132 (67%)	0.94
		A/G	48 (28%)	56 (28%)	
		G/G	9 (5%)	9 (5%)	
		A	272	320	0.7
		G	66	74	
***STK39***	*rs6749447*	G/G	24 (15%)	38 (19%)	0.16
		T/G	69 (42%)	90 (46%)	
		T/T	72 (44%)	67 (34%)	
		G	117	166	0.05
		T	213	224	
***BDKRB2***	*rs8012552*	C/C	28 (17%)	30 (15%)	0.34
		C/T	73 (43%)	100 (51%)	
		T/T	68 (4%)	67 (34%)	
		C	129	160	0.5
		T	209	234	
	*rs1799722*	C/C	63 (37%)	76 (39%)	0.7
		T/C	82 (49%)	88 (45%)	
		T/T	24 (14%)	33 (17%)	
		C	208	240	0.86
		T	130	154	
***KCNJ1***	*rs11600347*	C/C	143 (85%)	171 (87%)	0.41
		C/A	26 (15%)	25 (13%)	
		A/A	0 (0%)	1 (1%)	
		C	312	367	0.66
		A	26	27	
	*rs12795437*	G/G	141 (86%)	172 (87%)	0.48
		G/C	23 (14%)	24 (12%)	
		C/C	0 (0%)	1 (1%)	
		G	305	368	0.83
		C	23	26	
	*rs59172778*	Monomorphic SNP			
	*rs675388*	G/G	130 (77%)	145 (74%)	0.68
		G/A	37 (22%)	48 (24%)	
		A/A	2 (1%)	4 (2%)	
		G	297	338	0.41
		A	41	56	
***WNK1***	*rs880054*	C/C	59 (36%)	49 (25%)	
		C/T	79 (48%)	99 (51%)	0.049
		T/T	28 (17%)	48 (24%)	
		C	197	197	0.01
		T	135	195	
***LUC7L2***	*rs6947309*	C/C	77 (46%)	88 (45%)	0.7
		C/T	68 (40%)	86 (44%)	
		T/T	24 (14%)	23 (12%)	
		C	222	262	0.81
		T	116	132	
***NEDD4L***	*rs292449*	G/G	42 (26%)	56 (30%)	0.71
		C/G	85 (52%)	92 (48%)	
		C/C	36 (22%)	42 (22%)	
		G	169	204	0.62
		C	157	176	
	*rs75982813*	A/A	156 (93%)	179 (91%)	0.49
		G/A	12 (7%)	18 (9%)	
		A	324	376	0.5
		G	12	18	
***NPHS1***	*rs3814995*	C/C	102 (60%)	118 (60%)	0.8
		C/T	55 (33%)	68 (35%)	
		T/T	12 (07%)	11 (06%)	
		C	259	304	0.86
		T	79	90	
***CACNA1C***	*rs2239128*	C/C	53 (32%)	79 (40%)	0.22
		C/T	84 (50%)	86 (44%)	
		T/T	31 (18%)	31 (16%)	
		C	190	244	0.12
		T	146	148	
	*rs2238032*	G/G	1 (1%)	0 (0%)	0.11
		T/G	6 (4%)	2 (1%)	
		T/T	159 (96%)	194 (99%)	
		T	324	390	0.05
		G	8	2	
	*rs1051375*	A/A	35 (21%)	55 (28%)	0.28
		G/A	90 (53%)	95 (48%)	
		G/G	44 (26%)	47 (24%)	
		G	178	189	0.21
		A	160	205	

Significant *p*-values are considered as significant at *p* < 0.05. NA: Not available. *p*-values < 0.003 (0.05/# of SNPs, 0.05/15 = 0.003 after applying multiple comparisons) are considered significant.

**Table 5:** Genetic models and distributions of the SNPs within the genes in hypertensive patients and controls.

Gene	SNP_ID	Model	Genotype	Controls N (%)	Cases N (%)	OR (95% CI)	*p*-Value
***NPHS1***	*rs3814995*	Codominant	C/C	102 (60.4%)	118 (59.9%)	1	0.8
			C/T	55 (32.5%)	68 (34.5%)	1.07 (0.69–1.66)	
			T/T	12 (7.1%)	11 (5.6%)	0.79 (0.34–1.87)	
		Dominant	C/C	102 (60.4%)	118 (59.9%)	1	0.93
			C/T-T/T	67 (39.6%)	79 (40.1%)	1.02 (0.67–1.55)	
		Recessive	C/C-C/T	157 (92.9%)	186 (94.4%)	1	0.55
			T/T	12 (7.1%)	11 (5.6%)	0.77 (0.33–1.80)	
		Overdominant	C/C-T/T	114 (67.5%)	129 (65.5%)	1	0.69
			C/T	55 (32.5%)	68 (34.5%)	1.09 (0.71–1.69)	
***STK39***	*rs6749447*	Codominant	T/T	72 (43.6%)	67 (34.4%)	1	0.16
			G/T	69 (41.8%)	90 (46.1%)	1.40 (0.89–2.21)	
			G/G	24 (14.6%)	38 (19.5%)	1.70 (0.92–3.13)	
		Dominant	T/T	72 (43.6%)	67 (34.4%)	1	0.072
			G/T-G/G	93 (56.4%)	128 (65.6%)	1.48 (0.97–2.27)	
		Recessive	T/T-G/T	141 (85.5%)	157 (80.5%)	1	0.21
			G/G	24 (14.6%)	38 (19.5%)	1.42 (0.81–2.49)	
		Overdominant	T/T-G/G	96 (58.2%)	105 (53.9%)	1	0.41
			G/T	69 (41.8%)	90 (46.1%)	1.19 (0.78–1.81)	
***NEDD4L***	*rs292449*	Codominant	G/G	42 (25.8%)	56 (29.5%)	1	0.71
			C/G	85 (52.1%)	92 (48.4%)	0.81 (0.49–1.33)	
			C/C	36 (22.1%)	42 (22.1%)	0.88 (0.48–1.59)	
		Dominant	G/G	42 (25.8%)	56 (29.5%)	1	0.44
			C/G-C/C	121 (74.2%)	134 (70.5%)	0.83 (0.52–1.33)	
		Recessive	G/G-C/G	127 (77.9%)	148 (77.9%)	1	1
			C/C	36 (22.1%)	42 (22.1%)	1.00 (0.60–1.66)	
		Overdominant	G/G-C/C	78 (47.9%)	98 (51.6%)	1	0.49
			C/G	85 (52.1%)	92 (48.4%)	0.86 (0.57–1.31)	
	*rs75982813*	Codominant	A/A	156 (92.9%)	179 (90.9%)	1	0.49
			G/A	12 (7.1%)	18 (9.1%)	1.31 (0.61–2.80)	
***KCNJ1***	*rs11600347*	Codominant	C/C	143 (84.6%)	171 (86.8%)	1	0.41
			C/A	26 (15.4%)	25 (12.7%)	0.80 (0.44–1.45)	
			A/A	0 (0%)	1 (0.5%)	0.00 (0.00–NA)	
		Dominant	C/C	143 (84.6%)	171 (86.8%)	1	0.55
			C/A-A/A	26 (15.4%)	26 (13.2%)	0.84 (0.46–1.50)	
		Recessive	C/C-C/A	169 (100%)	196 (99.5%)	1	0.27
			A/A	0 (0%)	1 (0.5%)	0.00 (0.00–NA)	
		Overdominant	C/C-A/A	143 (84.6%)	172 (87.3%)	1	0.46
			C/A	26 (15.4%)	25 (12.7%)	0.80 (0.44–1.45)	
	*rs12795437*	Codominant	G/G	141 (86%)	172 (87.3%)	1	0.48
			G/C	23 (14%)	24 (12.2%)	0.86 (0.46–1.58)	
			C/C	0 (0%)	1 (0.5%)	0.00 (0.00–NA)	
		Dominant	G/G	141 (86%)	172 (87.3%)	1	0.71
			G/C-C/C	23 (14%)	25 (12.7%)	0.89 (0.48–1.64)	
		Recessive	G/G-G/C	164 (100%)	196 (99.5%)	1	0.27
			C/C	0 (0%)	1 (0.5%)	0.00 (0.00–NA)	
		Overdominant	G/G-C/C	141 (86%)	173 (87.8%)	1	0.61
			G/C	23 (14%)	24 (12.2%)	0.85 (0.46–1.57)	
	*rs59172778*	---------------------------------Monomorphic SNP----------------------------					
	*rs675388*	Codominant	G/G	130 (76.9%)	145 (73.6%)	1	0.68
			G/A	37 (21.9%)	48 (24.4%)	1.16 (0.71–1.90)	
			A/A	2 (1.2%)	4 (2%)	1.79 (0.32–9.95)	
		Dominant	G/G	130 (76.9%)	145 (73.6%)	1	0.46
			G/A-A/A	39 (23.1%)	52 (26.4%)	1.20 (0.74–1.93)	
		Recessive	G/G-G/A	167 (98.8%)	193 (98%)	1	0.52
			A/A	2 (1.2%)	4 (2%)	1.73 (0.31–9.57)	
		Overdominant	G/G-A/A	132 (78.1%)	149 (75.6%)	1	0.58
			G/A	37 (21.9%)	48 (24.4%)	1.15 (0.71–1.87)	
***WNK1***	*rs880054*	Codominant	C/C	59 (35.5%)	49 (25%)	1	0.049
			C/T	79 (47.6%)	99 (50.5%)	1.51 (0.93–2.44)	
			T/T	28 (16.9%)	48 (24.5%)	2.06 (1.13–3.76)	
		Dominant	C/C	59 (35.5%)	49 (25%)	1	0.029
			C/T-T/T	107 (64.5%)	147 (75%)	1.65 (1.05–2.60)	
		Recessive	C/C-C/T	138 (83.1%)	148 (75.5%)	1	0.074
			T/T	28 (16.9%)	48 (24.5%)	1.60 (0.95–2.69)	
		Overdominant	C/C-T/T	87 (52.4%)	97 (49.5%)	1	0.58
			C/T	79 (47.6%)	99 (50.5%)	1.12 (0.74–1.70)	
***LUC7L2***	*rs6947309*	Codominant	C/C	77 (45.6%)	88 (44.7%)	1	0.7
			C/T	68 (40.2%)	86 (43.6%)	1.11 (0.71–1.72)	
			T/T	24 (14.2%)	23 (11.7%)	0.84 (0.44–1.60)	
		Dominant	C/C	77 (45.6%)	88 (44.7%)	1	0.86
			C/T-T/T	92 (54.4%)	109 (55.3%)	0.96 (0.64–1.46)	
		Recessive	C/C-C/T	145 (85.8%)	174 (88.3%)	1	0.47
			T/T	24 (14.2%)	23 (11.7%)	1.25 (0.68–2.31)	
		Overdominant	C/C-T/T	101 (59.8%)	111 (56.4%)	1	0.51
			C/T	68 (40.2%)	86 (43.6%)	1.04 (0.69–1.57)	
***NPPA***	*rs5065*	Codominant	A/A	112 (66.3%)	132 (67%)	1	0.95
			A/G	48 (28.4%)	56 (28.4%)	0.99 (0.62–1.57)	
			G/G	9 (5.3%)	9 (4.6%)	0.85 (0.33–2.21)	
		Dominant	A/A	112 (66.3%)	132 (67%)	1	0.88
			A/G-G/G	57 (33.7%)	65 (33%)	0.97 (0.63–1.50)	
		Recessive	A/A-A/G	160 (94.7%)	188 (95.4%)	1	0.74
			G/G	9 (5.3%)	9 (4.6%)	0.85 (0.33–2.20)	
		Overdominant	A/A-G/G	121 (71.6%)	141 (71.6%)	1	1
			A/G	48 (28.4%)	56 (284%)	1.00 (0.63–1.58)	
***BDKRB2***	*rs8012552*	Codominant	T/T	68 (40.2%)	67 (34%)	1	0.34
			C/T	73 (43.2%)	100 (50.8%)	1.39 (0.88–2.19)	
			C/C	28 (16.6%)	30 (15.2%)	1.09 (0.59–2.01)	
		Dominant	T/T	68 (40.2%)	67 (34%)	1	0.22
			C/T-C/C	101 (59.8%)	130 (66%)	1.31 (0.85–2.00)	
		Recessive	T/T-C/T	141 (83.4%)	167 (84.8%)	1	0.73
			C/C	28 (16.6%)	30 (15.2%)	0.90 (0.52–1.59)	
		Overdominant	T/T-C/C	96 (56.8%)	97 (49.2%)	1	0.15
			C/T	73 (43.2%)	100 (50.8%)	1.36 (0.90–2.05)	
	*rs1799722*	Codominant	C/C	63 (37.3%)	76 (38.6%)	1	0.7
			T/C	82 (48.5%)	88 (44.7%)	0.89 (0.57–1.39)	
			T/T	24 (14.2%)	33 (16.8%)	1.14 (0.61–2.12)	
		Dominant	C/C	63 (37.3%)	76 (38.6%)	1	0.8
			T/C-T/T	106 (62.7%)	121 (61.4%)	0.95 (0.62–1.45)	
		Recessive	C/C-T/C	145 (85.8%)	164 (83.2%)	1	0.5
			T/T	24 (14.2%)	33 (16.8%)	1.22 (0.69–2.15)	
		Overdominant	C/C-T/T	87 (51.5%)	109 (55.3%)	1	0.46
			T/C	82 (48.5%)	88 (44.7%)	0.86 (0.57–1.29)	
***CACNA1C***	*rs2239128*	Codominant	C/C	53 (31.6%)	79 (40.3%)	1	0.22
			T/C	84 (50%)	86 (43.9%)	1.46 (0.92–2.31)	
			T/T	31 (18.4%)	31 (15.8%)	1.49 (0.81–2.74)	
		Dominant	C/C	53 (31.6%)	79 (40.3%)	1	0.082
			T/C-T/T	115 (68.5%)	117 (59.7%)	1.47 (0.95–2.26)	
		Recessive	C/C-T/C	137 (81.5%)	165 (84.2%)	1	0.51
			T/T	31 (18.4%)	31 (15.8%)	1.20 (0.70–2.08)	
		Overdominant	C/C-T/T	84 (50%)	110 (56.1%)	1	0.24
			T/C	84 (50%)	86 (43.9%)	1.28 (0.85–1.93)	
	*rs2238032*	Codominant	T/T	159 (95.8%)	194 (99%)	1	0.11
			G/T	6 (3.6%)	2 (1%)	0.27 (0.05–1.37)	
			G/G	1 (0.6%)	0 (0%)	0.00 (0.00–NA)	
		Dominant	T/T	159 (95.8%)	194 (99%)	1	0.05
			G/T-G/G	7 (4.2%)	2 (1%)	0.23 (0.05–1.14)	
		Recessive	T/T-G/T	165 (99.4%)	196 (100%)	1	0.21
			G/G	1 (0.6%)	0 (0%)	0.00 (0.00–NA)	
		Overdominant	T/T-G/G	160 (96.4%)	194 (99%)	1	0.09
			G/T	6 (3.6%)	2 (1%)	0.27 (0.05–1.38)	
	*rs1051375*	Codominant	G/G	44 (26%)	47 (23.9%)	1	0.28
			G/A	90 (53.2%)	95 (48.2%)	1.01 (0.61–1.67)	
			A/A	35 (20.7%)	55 (27.9%)	0.68 (0.38–1.23)	
		Dominant	G/G	44 (26%)	47 (23.9%)	1	0.63
			G/A-A/A	125 (74%)	150 (76.1%)	0.89 (0.55–1.43)	
		Recessive	G/G-G/A	134 (79.3%)	142 (72.1%)	1	0.11
			A/A	35 (20.7%)	55 (27.9%)	0.67 (0.42–1.10)	
		Overdominant	G/G-A/A	79 (46.8%)	102 (51.8%)	1	0.34
			G/A	90 (53.2%)	95 (48.2%)	1.22 (0.81–1.85)	

Significant *p*-values are considered significant at *p* < 0.05. *p*-values < 0.003 (0.05/# of SNPs, 0.05/15 = 0.003 after applying multiple comparisons) are considered significant. NA: Not available.

**Table 6:** Summary of the genetic association analysis results for hypertension susceptibility.

Gene	SNPs	Genetic Model	Significant Finding	Effect on HTN	*p*-Value	Odds Ratio (OR)
***WNK1***	*rs880054*	Codominant	Individuals with the TT genotype are 2.06 times more likely to have hypertension compared to individuals with the CC genotype.	Increase Risk of HTN	0.049	2.06
		Dominant	Individuals with at least one T allele (genotype TT or CT) are 1.6 times more likely to have hypertension compared to individuals with the CC genotype.	Increase Risk of HTN	0.029	1.65

**Table 7:** Haplotype analysis of the *KCNJ1*, *NEDD4L*, and *BDKRB2* gene variants among the hypertensive patients and healthy controls.

Gene	Haplotypes	Frequency		Odd Ratio (95% CI)	*p*-Value
		Controls	Cases		
***KCNJ1***	**C** **G** **G**	0.8018	0.7973	1	---
	**C** **G** **A**	0.1213	0.1341	1.18 (0.77–1.83)	0.45
	**A** **C** **G**	0.068	0.058	0.97 (0.53–1.75)	0.91
***NEDD4L***	**G** **A**	0.4957	0.5059	1	---
	**C** **A**	0.4686	0.4484	0.94 (0.69–1.27)	0.67
	**G** **G**	0.0229	0.0307	1.36 (0.37–5.00)	0.65
***BDKRB2***	**T** **C**	0.3641	0.369	1	---
	**C** **C**	0.2513	0.2402	0.94 (0.60–1.47)	0.8
	**T** **T**	0.2542	0.2249	0.87 (0.55–1.37)	0.54
	**C** **T**	0.1304	0.1659	1.23 (0.78–1.93)	0.38
***CACNA1C***	**C** **T** **A**	0.4165	0.4728	1	---
	**T** **T** **G**	0.3534	0.3295	0.80 (0.58–1.12)	0.19
	**C** **T** **G**	0.1491	0.1452	0.85 (0.55–1.33)	0.48
	**T** **T** **A**	0.0569	0.0474	0.75 (0.35–1.58)	0.45

Significant *p*-values are considered substantial at *p* < 0.05.

## Data Availability

The data used to support the findings of this study are available from the corresponding author upon request.

## Supplementary Materials

The following supporting information can be downloaded at: https://www.mdpi.com/article/10.3390/medicina61010156/s1, Table S1: Clinical characteristic of hypertensive patient; Table S2: Association between different STK39, CACNA1C, WNK1, LUC7L2, KCNJ1, NEDD4L and NPHS1 SNPs and the clinical characteristics of HTN patients.
