# Supplementary material for: Genomic Insights into Blood Pressure Regulation: Exploring Ion Channel and Transporter Gene Variations in Jordanian Hypertensive Individuals
Source: Medicina (Kaunas). 2025 Jan 17;61(1):156. doi: 10.3390/medicina61010156 (PMC11766771; doi:10.3390/medicina61010156)
Supplement: Supplementary file 1 [file medicina-61-00156-s001.zip › medicina-3370344-supplementary.pdf]

Supplementary Table S1. Clinical characteristics of hypertensive patients.

Clinical characteristics	Frequency N (%)	
**Gender**	Male	115 (57.5%)
	Female	85 (42.5%)
**Known HTN**	Yes	186 (93%)
	No	14 (7%)
**Newly diagnosed**	Yes	14 (7%)
	No	186 (93%)
**Number of years of HTN**	<10	108 (54%)
	≥10	92 (46%)
**DM**	Yes	111 (55.5%)
	No	89 (44.5%)
**Number of years of DM**	<10	43 (38.7%)
	≥10	68 (61.3%)
**IHD**	Yes	104 (52%)
	No	96 (48%)
**Heart failure**	HfpEF	162 (81%)
	HfrEF	12 (6%)
	HfmrEF	26 (13%)
**EF**	(50-70)%	161 (80.5%)
	(41-49)%	23 (11.5%)
	≤40	16 (8%)
**Peripheral vascular disease**	Yes	60 (30%)
	No	140 (70%)
**Cerebrovascular accident**	Yes	11 (5.5%)
	No	189 (94.5%)
**Chronic kidney disease**	Yes	11 (5.5%)
	No	189 (94.5%)
**Dialysis**	Yes	19 (9.5%)
	No	181 (90.5%)
**Atrial fibrillation**	Yes	11 (5.5%)
	No	189 (94.5%)
**Smoker**	Yes	56 (28%)
	No	144 (72%)

**Ex-smoker**	Yes	33 (16.5%)
	No	167 (83.5%)
**Diet**	Yes	62 (31%)
	No	138 (69%)
**Exercise**	Yes	35 (17.5%)
	No	165 (82.5%)
**Years of smoking**	<30	17(30.3%)
	≥30	39 (69.6%)
**SBP**	<90 mmHg	1 (0.5%)
	(90-120) mmHg	44 (22%)
	>120 mmHg	155 (77.5%)
**DBP**	<60 mmHg	5 (2.5%)
	(60-80) mmHg	118 (59%)
	>80 mmHg	77 (38.5%)
**PULS RATE**	<60 bpm	9 (4.5%)
	60-100 bpm	175 (87.5%)
	>100 bpm	16 (8%)
**Thiazide Diuretics mg per day**	Yes	99 (49.5%)
	No	101 (50.5%)
**B-blocker mg per day**	Yes	153 (76.5%)
	No	47 (23.5%)
**ACEi mg per day**	Yes	59 (29.5%)
	No	141 (70.5%)
**CCB mg per day**	Yes	87 (43.5%)
	No	113 (56.5%)
**ARB mg per day**	Yes	75 (37.5%)
	No	125 (62.5%)
**Number of required HTN drug**	1	35 (17.5%)
	2	75 (37.5%)
	3	40 (20%)
	4	27 (13.5%)
	5	3 (0.5%)
**Other HTN medications from other classes**	Yes	14 (7%)
	No	186 (93%)
**NSAID/COX2**	Yes	157 (78.5%)

	No	43 (21.5%)
**Antiplatelet**	Yes	84 (42%)
	No	116 (58%)
**Other medications**	Yes	46 (23%)
	No	154 (77%)
**Cardiovascular medications**	Yes	147 (73.5%)
	No	53 (26.5%)
**Other stomach drug**	Yes	139 (69.5%)
	No	61 (30.5%)
**Other osteoporosis**	Yes	33 (16.5%)
	No	167 (83.5%)
**Anticonvulsant**	Yes	13 (6.5%)
	No	187 (93.5%)
**Other for chest pain (angina)**	Yes	14 (7%)
	No	186 (93%)
**HB**	<12 (g/dL)	64 (32%)
	12-16 (g/dL)	119 (59.5%)
	>16 (g/dL)	17 (8.5%)
**WBC**	$<4 \times 10^3/\mu L$	8 (4%)
	$(4\text{-}11)\times 10^3/\mu L$	162 (81%)
	$>11 \times 10^3/\mu L$	30 (15%)
**PLT**	$<150 \times 10^3/\mu L$	14 (7%)
	$(150\text{-}459) \times 10^3/\mu L$	181 (90.5%)
	$>450 \times 10^3/\mu L$	5 (2.5%)
**Na**	<135 (mEq/L)	14 (7%)
	135-145 (mEq/L)	172 (86%)
	>145 (mEq/L)	14 (7%)
**K**	<3.5 (mEq/L)	4 (2%)
	3.5-5 (mEq/L)	160 (80%)
	>5 (mEq/L)	36 (18%)
**Urea**	<5 (mg/dL)	75 (37.5%)
	5-20 (mg/dL)	110 (55%)
	>20 (mg/dL)	15 (7.5%)
**Creatinine**	<61 (μmol/L)	24 (12%)
	61-114 (μmol/L)	125 (62.5%)

	>114 (µmol/L)	51 (25.5%)
**LDL**	<2.6 (mmol/L)	97 (64.2%)
	2.6-3.4 (mmol/L)	29 (19.2%)
	>3.4 (mmol/L)	25 (16.6%)
**HDL**	< 1.30 (mmol/L)	114 (75.5%)
	1.3-1.53 (mmol/L)	22 (14.6%)
	> 1.53 (mmol/L)	15 (9.9%)
**T. GLYCERIDE**	<1.7(mmol/L)	54 (36.2%)
	1.7-2.3 (mmol/L)	43 (28.8%)
	>2.3 (mmol/L)	52 (34.8%)
**T. Protein**	<60 (g/L)	11 (6.4%)
	60-83 (g/L)	151 (88.3%)
	>83 (g/L)	9 (5.3%)
**Albumin**	<3.4 (g/dL)	23 (13.4%)
	3.4-5.4 (g/dL)	147 (85.5%)
	>5.4 (g/dL)	2 (1.1%)

Supplementary table S2. Association between different *STK39, CACNA1C, WNK1, LUC7L2*, *KCNJ1*, *NEDD4L* and *NPHS1* SNPs and the clinical characteristics of HTN patients.

**Clinical characteristics**	***STK39***	***BDKRB2***		***CACNA1C***			***WNK1***	***LUC7L2***	***KCNJ1***			***NEDD4L***		***NPHS1***
	**rs6749447**	**rs1799722**	**rs8012552**	**rs2239128**	**rs2238032**	**rs1051375**	**rs880054**	**rs6947309**	**rs675388**	**rs12795437**	**rs11600347**	**rs75982813**	**rs292449**	**rs3814995**
**Age**	NA	0.719[b]	0.128[b]	1.893[b]	NA	0.336[b]	0.451[b]	0.507[b]	0.399[b]	0.123[b]	0.196[b]	0.569[b]	1.724[b]	1.451[b]
		0.489	0.88	0.153		0.715	0.638	0.606	0.672	0.885	0.822	0.452	0.181	0.237
**Height**	0.927[b]	0.936[b]	5.996[b]	0.189[b]	NA	3.427[b]	0.946[b]	0.676[b]	1.552[b]	0.365[b]	0.335[b]	0.180[b]	0.730[b]	4.590[b]
	0.606	0.394	**0.003***	0.828		**0.034***	0.39	0.51	0.214	0.695	0.716	0.672	0.483	**0.011***
**Wight**	1.421[b]	0.022[b]	0.709[b]	0.014[b]	NA	0.398[b]	3.815[b]	0.713[b]	0.448[b]	0.170[b]	0..202[b]	0.347[b]	0.923[b]	0.495[b]
	0.061	0.978	0.493	0.986		0.672	**0.024***	0.492	0.639	0.844	0.817	0.557	0.399	0.61
**Gender**	1.500[a]	0.483[a]	2.234[a]	0.764[a]	0.063[a]	2.407[a]	1.597[a]	0..757[a]	4.774[a]	3.907[ab]	4.469[a]	0.572[a]	1.985[a]	10.683[a]
	0.472	0.785	0.327	0.682	0.802	0.3	0.45	0.685	0.092	0.142	0.107	0.449	0.371	**0.005***
**Known HTN**	0.168[a]	0.971[a]	0.996[a]	0.230[a]	0.155[a]	1.397[a]	5.178[a]	2.287[a]	1.280[a]	0.136[a]	0.109[a]	0.481[a]	1.706[a]	1.843[a]
	0.92	0.615	0.608	0.891	0.693	0.497	0.075	0.319	0.527	0.934	0.947	0.488	0.426	0.398
**Newly diagnosed**	0.168[a]	0.971[a]	0.996[a]	0.230[a]	0.155[a]	1.397[a]	5.178[a]	2.287[a]	1.280[a]	0.136[a]	0.109[a]	0.481[a]	1.706[a]	1.843[a]
	0.92	0.615	0.608	0.891	0.693	0.497	0.075	0.319	0.527	0.934	0.947	0.488	0.426	0.398
**Number of years of HTN**	1.474[b]	3.138[b]	0.237[b]	2.289[b]	NA	0.646[b]	0.800[b]	0.259[b]	0.637[b]	0.212[b]	0.197[b]	2.171[b]	0.160[b]	0.111[b]
	**0.044***	**0.046***	0.789	0.104		0.525	0.451	0.772	0.53	0.809	0.821	0.142	0.852	0.895
**DM**	0.831[a]	0.167[a]	3.083[a]	0.270[a]	1.646[a]	2.095[a]	0.814[a]	4.838[a]	1.069[a]	1.519[a]	1.167[a]	0.861[a]	0.115[a]	0.688[a]
	0.66	0.92	0.214	0.874	0.199	0.351	0.666	0.089	0.586	0.468	0.558	0.353	0.944	0.709
**DM Treatment**	69.943[a]	55.020[a]	63.612[a]	68.451[a]	50.579[a]	68.861[a]	57.510[a]	50.413[a]	46.827[a]	45.840[a]	43.976[a]	1.619[a]	7.064[a]	65.488[a]
	0.1	0.512	0.226	0.123	**0.006***	0.059	0.419	0.685	0.804	0.832	0.878	0.951	0.719	0.181
**Number of years of DM**	1.422[b]	0.688[b]	1.986[b]	0.219[b]	NA	0.064[b]	0.740[b]	1.055[b]	0.711[b]	3.245[b]	2.761[b]	0.052[b]	0.889[b]	0.448[b]
	0.061	0.504	0.14	0.804		0.938	0.479	0.35	0.492	**0.041***	0.066	0.819	0.413	0.639
**IHD**	1.563[a]	1.514[a]	0.181[a]	1.258[a]	0.002[a]	0.206[a]	6.466[a]	0.633[a]	6.045[a]	1.413[a]	1.200[a]	0.369[a]	3.330[a]	6.308[a]
	0.458	0.469	0.913	0.533	0.965	0.902	**0.039**	0.729	**0.049***	0.493	0.549	0.543	0.189	**0.043***
**Heart failure**	3.828[a]	18.256[a]	5.031[a]	6.345[a]	2.599[a]	1.526[a]	6.176[a]	3.029[a]	4.931[a]	5.244[a]	3.870[a]	3.171[a]	7.926[a]	10.814[a]

	0.872	**0.019**[*]	0.754	0.609	0.627	0.992	0.628	0.933	0.765	0.731	0.869	0.53	0.441	0.212
**EF**	30.944[a]	82.177[a]	54.215[a]	57.592[a]	32.783[a]	57.566[a]	69.413[a]	50.601[a]	73.580[a]	49.486[a]	50.139[a]	28.457[a]	54.877[a]	50.440[a]
	0.999	**0.03**[*]	0.686	0.564	0.332	0.491	0.19	0.801	0.112	0.832	0.814	0.546	0.592	0.806
**peripheral vascular disease**	1.456[a]	1.764[a]	0.977[a]	0.254[a]	0.404[a]	2.216[a]	0.308[a]	2.411[a]	2.091[a]	2.409[a]	2.425[a]	2.146[a]	2.150[a]	0.163[a]
	0.483	0.414	0.613	0.881	0.525	0.33	0.857	0.3	0.352	0.3	0.297	0.143	0.341	0.922
**cerebrovascular accident**	0.512[a]	3.241[a]	2.001[a]	0.268[a]	0.120[a]	2.327[a]	7.800[a]	4.130[a]	0.526[a]	0.168[a]	0.201[a]	0.000[a]	3.152[a]	0.689[a]
	0.774	0.198	0.368	0.875	0.729	0.312	**0.02**[*]	0.127	0.769	0.92	0.905	0.995	0.207	0.708
**chronic kidney disease**	0.062[a]	0.031[a]	3.617[a]	4.941[a]	0.120[a]	1.554[a]	2.508[a]	0.566[a]	0.526[a]	17.537[a]	17.452[a]	1.172[a]	2.270[a]	4.239[a]
	0.969	0.985	0.164	0.085	0.729	0.46	0.285	0.754	0.769	**0.0002**[*]	**0.0002**[*]	0.279	0.321	0.12
**Dialysis**	2.568[a]	3.533[a]	0.102[a]	0.765[a]	0.217[a]	0.547[a]	0.687[a]	0.070[a]	1.177[a]	9.761[a]	9.677[a]	0.049[a]	0.377[a]	0.094[a]
	0.277	0.171	0.95	0.682	0.641	0.761	0.709	0.966	0.555	**0.008**[*]	**0.008**[*]	0.825	0.828	0.954
**Atrial fibrillation**	0.392[a]	0.677[a]	2.256[a]	0.093[a]	0.120[a]	3.538[a]	7.808[a]	1.528[a]	0.278[a]	1.693[a]	1.771[a]	1.172[a]	1.600[a]	4.703[a]
	0.822	0.713	0.324	0.955	0.729	0.171	**0.02**[*]	0.466	0.87	0.429	0.412	0.279	0.449	0.095
**Smoker**	1.728[a]	2.620[a]	1.001[a]	0.555[a]	0.788[a]	0.319[a]	2.757[a]	3.486[a]	0.357[a]	6.930[a]	8.549[a]	1.246[a]	3.631[a]	1.032[a]
	0.421	0.27	0.606	0.758	0.375	0.852	0.252	0.175	0.837	**0.031**[*]	**0.014**[*]	0.264	0.163	0.597
**Ex-smoker**	3.556[a]	0.759[a]	0.105[a]	0.074[a]	1.587[a]	0.247[a]	0.794[a]	3.104[a]	11.104[a]	4.997[a]	4.997[a]	0.000[a]	0.529[a]	3.637[a]
	0.169	0.684	0.949	0.964	0.208	0.884	0.672	0.212	**0.004**[*]	0.082	0.082	0.992	0.768	0.162
**Diet**	4.488[a]	2.211[a]	0.118[a]	2.118[a]	0.358[a]	6.571[a]	0.148[a]	2.603[a]	0.547[a]	2.644[a]	2.827[a]	1.684[a]	4.079[a]	3.155[a]
	0.106	0.331	0.943	0.347	0.55	**0.037**[*]	0.929	0.272	0.761	0.267	0.243	0.194	0.13	0.206
**Exercise**	0.280[a]	0.887[a]	1.010[a]	1.697[a]	0.424[a]	0.052[a]	2.386[a]	0.851[a]	3.487[a]	3.726[a]	3.983[a]	0.016[a]	0.709[a]	0.140[a]
	0.869	0.642	0.603	0.428	0.515	0.974	0.303	0.653	0.175	0.155	0.136	0.898	0.701	0.932
**years of smoking**	1.246[b]	0.909[b]	0.776[b]	0.452[b]	NA	0.180[b]	2.065[b]	1.487[b]	0.402[b]	2.163[b]	3.231[b]	0.236[b]	2.241[b]	0.897[b]
	0.166	0.404	0.462	0.637		0.836	0.13	0.229	0.669	0.118	**0.042**[*]	0.628	0.106	0.41
**SBP**	0.912[b]	0.237[b]	0.335[b]	0.021[b]	NA	0.241[b]	3.666[b]	0.005[b]	2.438[b]	0.810[b]	0.747[b]	0.220[b]	1.035[b]	0.864[b]
	0.631	0.789	0.715	0.979		0.786	**0.027**[*]	0.995	0.09	0.447	0.476	0.64	0.357	0.423
**DBP**	0.985[b]	0.383[b]	0.304[b]	1.128[b]	NA	0.667[b]	1.877[b]	0.782[b]	0.828[b]	1.436[b]	1.303[b]	0.585[b]	0.481[b]	0.807[b]
	0.507	0.682	0.738	0.326		0.514	0.156	0.759	0.439	0.24	0.274	0.445	0.619	0.448
**PULS RATE**	1.117[b]	1.371[b]	2.286[b]	0.874[b]	NA	2.023[b]	0.169[b]	0.347[b]	0.364[b]	3.577[b]	2.670[b]	0.014[b]	6.692[b]	0.502[b]

	0.306	0.256	0.104	0.419		0.135	0.844	0.707	0.695	**0.03**[*]	0.072	0.907	**0.002**[*]	0.606
**Thiazide Diuretics mg per day**	60.097[a]	49.471[a]	59.800[a]	65.811[a]	100.657[a]	57.460[a]	66.468[a]	43.249[a]	48.399[a]	133.184[a]	131.727[a]	11.669[a]	18.816[a]	44.706[a]
	0.4	0.78	0.41	0.225	**0.003**[*]	0.218	0.208	0.926	0.811	**0.004**[*]	**0.004**[*]	0.998	0.93	0.900
**B-blocker mg per day**	10.578[a]	23.477[a]	16.435[a]	24.664[a]	32.205[a]	19.603[a]	12.139[a]	21.557[a]	39.697[a]	16.771[a]	15.726[a]	8.193[a]	11.603[a]	21.243[a]
	0.911	0.173	0.562	0.134	**0.003**[*]	0.356	0.84	0.252	**0.002**[*]	0.539	0.612	0.515	0.867	0.267
**ACEi mg per day**	0.581[a]	4.560[a]	1.784[a]	0.238[a]	0.380[a]	5.959[a]	5.072[a]	2.480[a]	6.937[a]	0.566[a]	0.479[a]	1.984[a]	2.558[a]	0.745[a]
	0.748	0.102	0.41	0.888	0.537	0.051	0.079	0.289	**0.031**[*]	0.754	0.787	0.159	0.278	0.689
**CCB mg per day**	36.188[a]	16.250[a]	33.585[a]	23.200[a]	0.608[a]	17.351[a]	25.120[a]	25.734[a]	11.946[a]	211.831[a]	211.079[a]	3.601[a]	11.719[a]	16.619[a]
	**0.015**[*]	0.701	**0.029**[*]	0.279	1.00	0.499	0.197	0.175	0.918	**0.005**[*]	**0.005**[*]	0.964	0.304	0.678
**ARB mg per day**	51.659[a]	27.898[a]	37.186[a]	42.195[a]	1.199[a]	25.125[a]	34.448[a]	41.470[a]	21.110[a]	213.324[a]	215.522[a]	52.617[a]	34.706[a]	22.717[a]
	**0.044**[*]	0.831	0.414	0.221	1.00	0.865	0.542	0.244	0.977	0.000	0.000	**0.0001**[*]	0.53	0.958
**Number of required HTN drug**	1.153[b]	0.322[b]	3.693[b]	4.892[b]	NA	1.135[b]	0.224[b]	0.549[b]	2.252[b]	1.645[b]	1.572[b]	0.439[b]	0.269[b]	0.346[b]
	0.261	0.725	**0.027**[*]	**0.008**[*]		0.323	0.8	0.578	0.108	0.196	0.21	0.508	0.764	0.708
**CLASSES IT TAKEN**	118.817[a]	47.478[a]	56.729[a]	46.222[a]	7.905[a]	44.500[a]	47.530[a]	60.425[a]	55.005[a]	128.209[a]	125.471[a]	18.504[a]	5.004[a]	36.972[a]
	0.000[*]	0.494	0.182	0.546	0.999	0.535	0.492	0.108	0.227	0.000	0.000	0.778	0.757	0.876
**number of HTN drug from all classes**	1.171[b]	0.268[b]	3.978	6.039[b]	NA	1.962[b]	0.012[b]	0.005[b]	2.606[b]	1.218[b]	1.126[b]	0.085[b]	0.123[b]	0.386[b]
	0.239	0.765	**0.02**[*]	**0.003**[*]		0.143	0.988	0.995	0.076	0.298	0.326	0.771	0.884	0.68
**other HTN medications from other classes**	3.922[a]	12.037[a]	5.392[a]	10.743[a]	0.242[a]	7.515[a]	4.101[a]	8.266[a]	16.202[a]	5.022[a]	4.815[a]	2.377[a]	0.326[a]	8.990[a]
	0.864	0.15	0.715	0.217	0.993	0.482	0.848	0.408	**0.04**[*]	0.755	0.777	0.667	0.243	0.343
**NSAID/COX2**	196.003[a]	2.329[a]	2.335[a]	8.585[a]	0.568[a]	2.933[a]	4.178[a]	2.511[a]	2.744[a]	4.122[a]	4.264[a]	0.424[a]	1.792[a]	1.262[a]
	**0.000**[*]	0.676	0.674	0.072	0.753	0.569	0.382	0.643	0.602	0.39	0.372	0.809	0.408	0.868
**Antiplatelet**	221.293[a]	59.896[a]	57.007[a]	53.303[a]	6.496[a]	38.021[a]	55.381[a]	81.709[a]	57.147[a]	31.022[a]	29.529[a]	38.436[a]	8.456[a]	81.134[a]
	**0.000**	0.622	0.720	0.827	1.00	0.848	0.77	0.067	0.715	1.00	1.00	0.201	0.864	0.073
**Other medications**	121.152[a]	55.204[a]	66.702[a]	39.318[a]	97.612[a]	36.630[a]	43.282[a]	68.020[a]	85.441[a]	224.913[a]	223.896[a]	13.514[a]	39.945[a]	36.938[a]
	**0.000**[*]	0.285	0.057	0.862	1.00	0.348	0.738	**0.046**[*]	**0.001**[*]	0.000	0.000	0.97	0.845	0.915
**cardiovascular medications**	5.149[a]	6.386[a]	6.969[a]	9.201[a]	0.730[a]	9.715[a]	5.363[a]	3.270[a]	3.192[a]	9.590[a]	9.796[a]	10.842[a]	20.593[a]	6.859[a]
	0.742	0.604	0.54	0.326	0.948	0.286	0.718	0.916	0.922	0.295	0.28	**0.028**[*]	0.113	0.552

**Chemotherapy**	196.000[a]	1.245[a]	0.975[a]	1.286[a]	0.010[a]	0.883[a]	0.985[a]	1.245[a]	0..360[a]	0.146[a]	0.153[a]	0.101[a]	NA	0.673[a]
	**0.00**[*]	0.537	0.614	0.526	0.919	0.643	0.611	0.537	0.835	0.93	0.926	0.751		0.714
**other stomach drug**	199.185[a]	6.672[a]	6.897[a]	11.055[a]	3.022[a]	8.247[a]	10.282[a]	11.612[a]	5.020[a]	105.586[a]	105.152[a]	0.459[a]	3.113[a]	17.365[a]
	**0.000**[*]	0.756	0.735	0.353	0.697	0.605	0.416	0.312	0.89	0.000	0.000	0.994	0.794	0.067
**other osteoporosis**	3.256[a]	7.355[a]	13.983[a]	7.999[a]	0.394[a]	6.992[a]	5.439[a]	4.010[a]	8.347[a]	11.739[a]	11.416[a]	1.889[a]	10.039[a]	7.401[a]
	0.917	0.499	0.082	0.434	0.983	0.538	0.71	0.856	0.4	0.163	0.179	0.756	0.262	0.494
**Anticonvulsant**	2.108[a]	4.558[a]	1.290[a]	3.942[a]	0.144[a]	1.860[a]	5.411[a]	4.975[a]	2.520[a]	2.776[a]	2.547[a]	0.203[a]	4.553[a]	1.435[a]
	0.716	0.336	0.863	0.414	0.931	0.762	0.248	0.29	0.641	0.596	0.636	0.903	0.336	0.838
**other for chest pain (angina)**	4.527[a]	7.314[a]	10.709[a]	7.410[a]	23.276[a]	2.939[a]	4.707[a]	7.732[a]	13.308[a]	2.191[a]	2.292[a]	1.516[a]	7.836[a]	5.439[a]
	0.807	0.503	0.219	0.493	**0.0001**[*]	0.983	0.788	0.46	102	0.975	0.971	0.824	0.45	0.71
**Antipsychotic**	1.751[a]	3.217[a]	1.960[a]	6.828[a]	0.021[a]	NA	3.990[a]	8.839[a]	0.725[a]	7.381[a]	2.292[a]	0.203[a]	4.603[a]	17.658[a]
	0.781	0.522	0.743	0.145	0.99		0.407	0.065	0.948	0.117	0.133	0.903	0.331	**0.001**[*]
**immune suppressive/allergies**	10.578[a]	16.645[a]	15.733[a]	19.313[a]	0.109[a]	15.988[a]	16.036[a]	19.960[a]	30.464[a]	222.257[a]	221.111[a]	10.850[a]	19.456[a]	11.640[a]
	0.911	0.548	0.611	0.373	1.00	0.314	0.59	0.335	**0.033**[*]	**0.002**[*]	**0.002**[*]	0.286	0.364	0.865
**muscle relax**	0.534[a]	1.221[a]	0.838[a]	0.879[a]	0.031[a]	4.137[a]	3.752[a]	0.760[a]	0.182[a]	0.443[a]	0.463[a]	0.306[a]	0.333[a]	2.040[a]
	0.766	0.543	0.658	0.644	0.859	0.126	0.153	0.684	0.193	0.801	0.793	0.58	0.847	0.361
**HB**	0.376[b]	1.481[b]	1.134[b]	0.929[b]	NA	1.226[b]	1.096[b]	1.283[b]	0.271[b]	0.228[b]	0.229[b]	0.070[b]	1.291[b]	0.703[b]
	1.00	0.23	0.324	0.397		0.296	0.336	0.28	0.763	0.796	0.796	0.792	0.277	0.497
**WBC**	1.586[b]	0.526[b]	0.267[b]	0.483[b]	NA	0.229[b]	0.253[b]	0.904[b]	0.165[b]	4.221[b]	4.088[b]	0.378[b]	0.348[b]	0.279[b]
	**0.021**[*]	0.592	0.766	0.618		0.796	0.776	0.406	0.848	**0.016**[*]	**0.018**[*]	0.539	0.706	0.757
**PLT**	0.933[b]	1.561[b]	0.450[b]	0.753[b]	NA	0.609[b]	2.636[b]	0.237[b]	0.297[b]	1.011[b]	0.661[b]	0.182[b]	0.244[b]	0.423[b]
	0.597	0.213	0.638	0.472		0.545	0.074	0.79	0.743	0.366	0.518	0.671	0.784	0.656
**Na**	0.530[b]	3.477[b]	1.929[b]	1.174[b]	NA	1.855[b]	1.086[b]	1.887[b]	0.633[b]	0.944[b]	0.862[b]	0.095[b]	0.911[b]	0.306[b]
	0.993	**0.033**[*]	0.148	0.311		0.159	0.34	0.154	0.532	0.391	0.424	0.758	0.404	0.737
**K**	1.260[b]	2.953[b]	0.450[b]	2.749[b]	NA	0.277[b]	0.613[b]	0.950[b]	0.043[b]	0.252[b]	0.265[b]	0.361[b]	0.135[b]	0.164[b]
	0.158	0.055	0.638	0.066		0.758	0.543	0.389	0.958	0.777	0.768	0.549	0.874	0.849
**Urea**	0.967[b]	1.714[b]	0.529[b]	0.801[b]	NA	0.626[b]	1.806[b]	0.745[b]	0.189[b]	0.287[b]	0.232[b]	1.783[b]	1.785[b]	0.787[b]
	0.538	0.183	0.59	0.45		0.536	0.167	0.476	0.828	0.751	0.794	0.183	0.171	0.457

**Creatinine**	1.198[b]	0.994[b]	0.089[b]	1.490[b]	NA	0.055[b]	0.083[b]	0.104[b]	1.226[b]	1.672[b]	1.555[b]	0.023[b]	0.193[b]	0.684[b]
	0.211	0.372	0.915	0.228		0.946	0.92	0.901	0.296	0.191	0.214	0.881	0.824	0.506
**T. Cholesterol**	1.568[b]	0.065[b]	1.774[b]	0.320[b]	NA	0.137[b]	1.975[b]	0.183[b]	2.315[b]	0.088[b]	0.088[b]	1.257[b]	0.921[b]	0.483[b]
	**0.032**[*]	0.937	0.173	0.727		0.872	0.142	0.833	0.102	0.916	0.916	0.264	0.4	0.618
**LDL**	0.371[b]	0.264[b]	0.814[b]	1.365[b]	NA	0.860[b]	0.888[b]	0.258[b]	0.367[b]	0.149[b]	0.149[b]	0.145[b]	1.092[b]	0.091[b]
	1.00	0.768	0.445	0.258		0.425	0.413	0.773	0.693	0.862	0.862	0.704	0.338	0.913
**HDL**	0.927[b]	0.874[b]	0.391[b]	1.210[b]	NA	1.244[b]	1.449[b]	0.389[b]	1.146[b]	3.287[b]	3.287[b]	0.206[b]	0.453[b]	0.683[b]
	0.600	0.419	0.677	0.301		0.291	0.238	0.679	0.321	**0.04**[*]	**0.04**[*]	0.651	0.636	0.506
**T. GLYCERIDE**	0.365[b]	1.272[b]	0.587[b]	0.992[b]	NA	0.256[b]	3.745[b]	0.194[b]	0.097[b]	0.610[b]	0.610[b]	0.042[b]	0.352[b]	0.935[b]
	1.00	0.283	0.557	0.373		0.775	**0.026**[*]	0.824	0.907	0.545	0.545	0.383	0.704	0.395
**GLUCOSE**	1.003[b]	2.324[b]	1.498[b]	0.545[b]	NA	1.038[b]	0.464[b]	1.019[b]	0.477[b]	1.062[b]	1.062[b]	0.081[b]	0.107[b]	0.103[b]
	0.478	0.101	0.226	0.581		0.356	0.63	0.363	0.622	0.348	0.348	0.777	0.898	0.902
**HBAC1**	0.892[b]	2.981[b]	0.539[b]	1.026[b]	NA	1.196[b]	1.980[b]	0.834[b]	0.588[b]	1.825[b]	1.825[b]	3.287[b]	0.168[b]	0.281[b]
	0.658p	**0.053**[*]	0.585	0.361		0.305	0.141	0.436	0.557	0.164	0.164	0.072	0.846	0.755
**Albumin**	1.045[b]	0.532[b]	0.543[b]	1.207[b]	NA	0.144[b]	1.738[b]	1.115[b]	0.353[b]	0.540[b]	0.540[b]	0.409[b]	0.920[b]	0.390[b]
	0.412	0.588	0.582	0.302		0.866	0.179	0.33	0.703	0.584	0.584	0.523	0.4	0.677
**T. Protein**	0.914[b]	2.333[b]	3.418[b]	0.106[b]	NA	2.786[b]	0.593[b]	2.071[b]	0.617[b]	4.018[b]	4.018[b]	0.461[b]	0.028[b]	0.224[b]
	0.628	0.1	**0.035**[*]	0.899		0.064	0.554	0.129	0.541	**0.02**[*]	**0.02**[*]	0.498	0.972	0.8
**LVH ON ECG**	4.557[a]	1.082[a]	1.004[a]	0.610[a]	1.860[a]	0.338[a]	1.233[a]	1.461[a]	7.381[ab]	0.808[a]	0.653[a]	3.582[a]	1.172[a]	2.749[a]
	0.102	0.582	0.605	0.737	0.173	0.844	0.54	0.482	**0.025**[*]	0.667	0.721	0.58	0.557	0.253
**LVH ON ECHO**	1.746[a]	2.290[a]	7.036[a]	1.168[a]	1.226[a]	5.596[a]	5.560[a]	2.130[a]	0.824[a]	1.671[a]	1.690[a]	1.988[a]	2.494[a]	0.620[a]
	0.418	0.318	**0.030**[*]	0.558	0.268	0.061	0.062	0.345	0.662	0.343	0.43	0.159	0.287	0.733
**creatinine clearance**	1.317[b]	0.835[b]	0.365[b]	0.081[b]	146.495[b]	0.353[b]	0.587[b]	0.260[b]	1.302[b]	0.823[b]	0.821[b]	0.184[b]	1.053[b]	2.282[b]
	0.113	0.435	0.964	0.922	**0.022**[*]	0.703	0.557	0.772	0.274	0.441	0.442	0.668	0.351	0.105

[a] Chi-squared value; Pearson's chi-squared test was used to determine genotype-phenotype association.

[b] F-value; Analysis of variance (ANOVA) test was used to determine genotype-phenotype association.

* $p<0.05$ was considered statistically significant.

P-values < 0.003 (0.05/# of SNPs, 0.05/15 = 0.003 after applying multiple comparison) are considered as significant.

NA: not available.
